# Temperature Self-Adaptive and Color-Adjustable Smart Window Based on Templated Cholesteric Liquid Crystals

**DOI:** 10.3390/polym16010082

**Published:** 2023-12-26

**Authors:** Changli Sun, Jiangang Lu

**Affiliations:** National Engineering Lab for TFT-LCD Materials and Technologies, School of Electronic Information and Electrical Engineering, Shanghai Jiao Tong University, Shanghai 200240, China; chlsun@sjtu.edu.cn

**Keywords:** cholesteric liquid crystals, smart window, template, self-adaptive

## Abstract

Cholesteric liquid crystals (CLCs) exhibit selective reflection due to their self-assembled helical superstructures. Reconfigurable templates can achieve integration functions via inducing processes of molecular assemblies. Here we demonstrate temperature self-adaptive and color-adjustable smart windows using CLCs, which are fabricated via the templating method and exhibit simultaneous reflections in the visible and infrared spectra. Reflection bands formed by the refilled CLC materials can be adjusted reversibly both upon thermal and electrical actuation. In CLC with adjustable reflection in the infrared, the central wavelength of the infrared reflection band can be adjusted from 950 nm to 1305 nm via temperature, and from 1150 nm to 950 nm via electric field. A temperature variation of 10.3 °C within 55 s was induced by the single-layer templated CLC cell, and a comfortable temperature range could be effectively maintained by the CLC cell in a varied environment. In CLC with dynamic color in the visible spectrum, color shifts from 530 nm to 650 nm tuned by temperature and from 530 nm to 440 nm adjusted by electric field were obtained. Temperature-responsive reflection in the infrared spectrum contributes to automatic thermal management, and electric-field-induced band shift in the visible spectrum enables active dynamic color adjustment. The presented templated CLC smart windows show considerable potential in energy conservation and biological clock regulation fields.

## 1. Introduction

CLCs are excellent candidates for application in many fields, such as displays, sensors, and filters, due to unique twist structures [1,2,3,4,5,6]. Their dynamic response to external stimuli, such as heat, light, or electricity, makes them attractive in smart windows [7,8,9,10,11]. Compared with traditional windows, smart windows enable dynamic regulation of daylighting, which is conducive to thermal management, energy consumption reduction, and color adjustment for humans [12,13,14,15,16,17]. According to Bragg’s law, CLCs in the planar orientation produce tunable selective reflection of incident light, covering the near-infrared band, whose energy accounts for most of the solar infrared radiation [18,19,20,21]. In addition, external stimuli are able to manipulate the color change of CLCs’ Bragg reflection, meeting the demands of decoration and antiglare [22]. Characteristics of CLC-based smart windows have been reported theoretically and experimentally. CLC films with broadband reflection for smart windows were prepared by doping ultraviolet absorption dyes [23,24], adding nanofiber films [25,26], and performing photomask polymerization [27]. Zhang et al. proposed the step-by-step light curing method, which formed a pitch gradient in the cell thickness direction, to develop a reflective broadband CLC film [28]. An electrically tunable infrared CLC reflector with negative dielectric liquid crystals (LCs) and long ethylene glycol twin crosslinkers was demonstrated [29]. CLCs with photocontrollable selective reflection band related to photoinduced E-Z isomerization of a photochromic mixture were prepared [30]. Zhang et al. prepared an electrically induced coloration CLC film with broadband reflection causing a temperature variation of 2.6 °C via polymerization-induced pitch gradient method [31]. D. Manaila-Maximean et al. reported polymer-dispersed CLCs reflecting in the infrared region using the photopolymerization-induced phase separation method [32]. A photoresponsive CLC composed of photoresponsive chiral photochromic dopant, non-photoresponsive chiral dopant, and nematic LCs was prepared, and the CLC exhibited color change between red and blue upon ultraviolet light irradiation [33]. A photochromic and thermochromic CLC of colorful patterns was achieved by synthesizing a chiral dopant with trans-p-hexoxycinnamic acid [34]. However, little has been studied about CLCs for temperature self-adaptive and color-adjustable smart windows of simultaneous bandgaps in the visible and infrared, and the adjustable capability especially in the infrared still needs to be improved.

Herein, tunable templated CLC films of simultaneous reflections in the visible and infrared for smart windows are proposed. Molecular assemblies of refilled materials are induced via the templating method and tuning performance of synchronous bandgaps upon thermal and electrical actuation is presented. The CLC bandgap in the infrared is able to spontaneously respond to temperature changes, which can reduce the building energy consumption through thermal control. In addition, band shift in the infrared upon electrical actuation is also illustrated. With a broad reflection band in the infrared, dynamic colors can be induced via changing temperature and applying an electric field. The presented CLCs for energy-saving and dynamic chromatic smart windows may find use in domains of intelligent buildings and transportation.

## 2. Design and Fabrication of Templated Single-Layer CLCs

To achieve simultaneous reflections in the visible and infrared spectra, two types of single-layer devices were designed and fabricated via the templating method—a templated CLC with adjustable reflection in the infrared spectrum and a templated CLC with dynamic color. The former was produced by refilling CLC materials of Bragg reflection in the infrared spectrum into a CLC template in the visible band, and the latter was made by filling CLCs with a bandgap in the visible spectrum into a CLC template of reflection in the infrared spectrum.

### 2.1. Material Preparation

**Figure 1:**
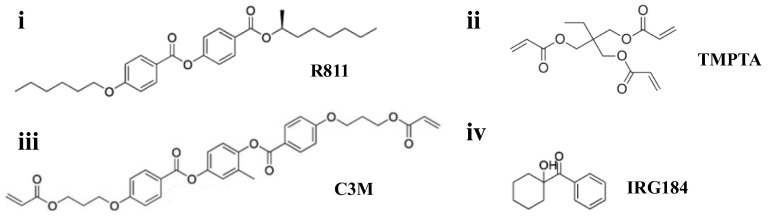
Molecular structures of chiral dopant, monomers, and photo-initiator. (**i**) R811, (**ii**) TMPTA, (**iii**) C3M, (**iv**) IRG184.

The single-layer CLC system was a combination of a polymer-stabilized CLC (PSCLC) template and a refilled CLC material with two reflection bands in the visible and infrared simultaneously. To fabricate CLC templates, we prepared PSCLC precursors of a positive nematic liquid crystal (HTD, n_e_ = 1.82, ∆n = 0.305, ∆ε = 7.9, Jiangsu Hecheng Display Technology Co., Ltd. (HCCH), Nanjing, China), a chiral dopant (R811, HTP (helical twisting power) = 10 µm^−1^, HCCH), an ultraviolet curable monomer (TMPTA, HCCH), a cross-linking agent (C3M), and a photo-initiator (IRG184, HCCH) (Figure 1). The refilled CLC material consisted of a nematic liquid crystal (HTD) and a chiral dopant (R811). The optical pitches of the PSCLC template and refilled CLC were different, with one located in the visible or infrared band, and the other in a second band. Five samples were fabricated to fulfill different performance requirements of heat management and color adjustment. Materials for the CLC samples of tunable selective reflection in the infrared band and template in the visible band are listed in Table 1. Components for the CLC samples of dynamic color in the visible band and template in the infrared band are shown in Table 2. The pitches of Sample 1, Sample 2, Sample 3, Sample 4, and Sample 5 were designed to be 317.8 nm, 569.7 nm, 689.7 nm, 689.7 nm, and 317.8 nm, respectively.

### 2.2. Device Fabrication

**Figure 2:**
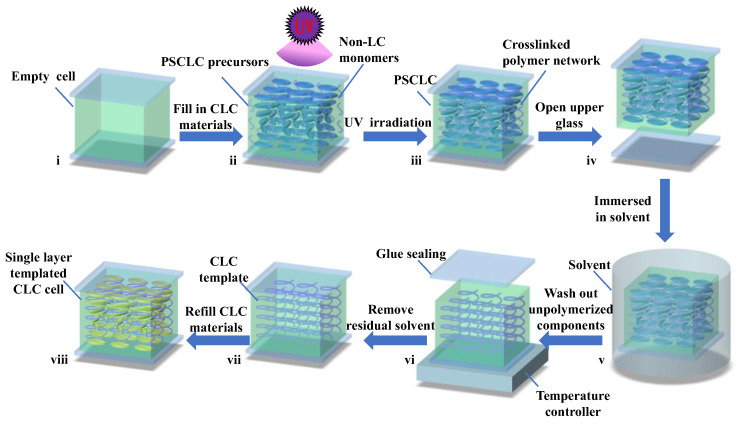
Schematic illustration of the CLC arrangements in the single-layer cell. (**i**) Empty cell, (**ii**) PSCLC precursors in cell under UV irradiation, (**iii**) PSCLC sample, (**iv**) opening the upper glass substrate of cell, (**v**) bottom substrate with PSCLC immersed in solvent, (**vi**) removing residual solvent and combining the upper substrate with bottom substrate with template, (**vii**) template of sample, (**viii**) cell after refilling material into the template.

The PSCLC precursors were injected into an empty cell of 20 µm gap with antiparallel alignment at the isotropic phase by capillary action (Figure 2i,ii). After being cured by ultraviolet irradiation (365 nm) with an intensity of 3.0 mw/cm^−2^ for 60 min at 50 °C, polymer monomers were crosslinked into a polymer network to stabilize the orientation of LC directors (Figure 2iii). And then the cell was separated into two substrates at 130 °C (Figure 2iv). One of the glass substrates close to the ultraviolet light was coated with the PSCLC film due to the ultraviolet irradiation penetrating from upper glass slide to bottom glass slide of the cell (Figure 2ii). The coated slide was then placed in a solution of ethanol and water to eliminate the unpolymerized components, containing LC, chiral dopant, unreacted monomers, and photo-initiator (Figure 2v). Addition of water to ethanol prevented deformation of the helical structure of the polymer template. The soaking time for the CLC template was determined by the weight ratio of ethanol. The coated slide was then heated on the temperature controller (HCS302, Instec Co., Boulder, CO, USA) at 50 °C for 30 min to evaporate the solvent (Figure 2vi). The glass slide was again glued onto the coated slide and exposed to ultraviolet light for 10 min to fabricate the single-layer cell (Figure 2vii). The CLC materials of LCs and chiral dopant were refilled into the single-layer cell at the isotropic phase until the cell was completely filled with the refilled materials (Figure 2viii). The fabrication process of the single-layer templated CLC with simultaneous reflections in the visible and infrared bands is demonstrated in Figure 2i–viii. This templating approach suggests routes for a fast template fabrication process.

**Figure 3:**
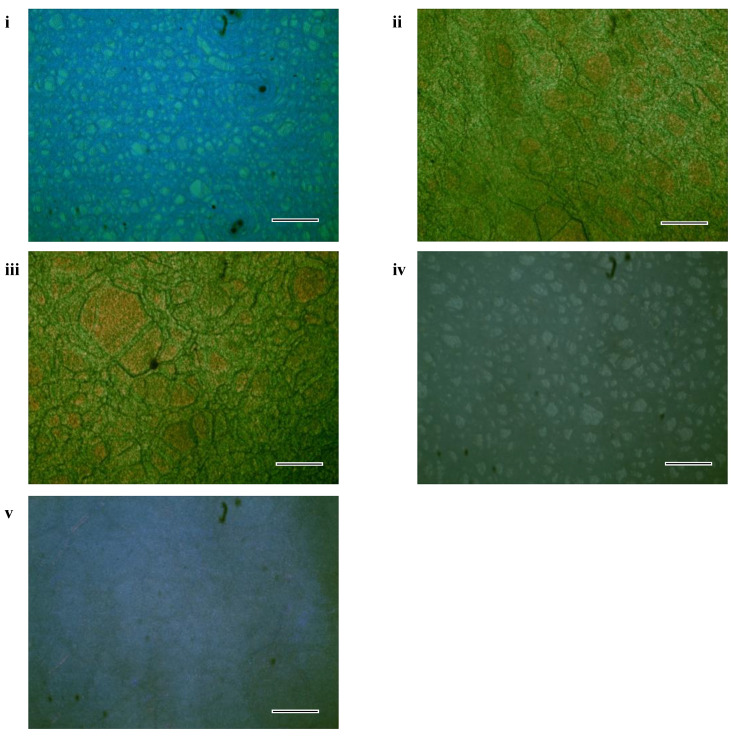
POM images showing platelet textures of (**i**) PSCLC of reflection in visible band after ultraviolet irradiation, (**ii**) Cell-1 after refilling Sample-2 into template of Sample-1, (**iii**) Cell-2 after refilling Sample-3 into template of Sample-1, (**iv**) PSCLC of bandgap in infrared band after ultraviolet irradiation, (**v**) Cell-3 after refilling Sample-5 into template of Sample-4. Scale bar, 100 µm.

Three single-layer templated CLC cells were fabricated to realize thermal management and color adjustment. Sample 1 with reflection in the visible band was filled into an empty cell at the isotropic phase (353 K), and then irradiated by ultraviolet light for 60 min at 50 °C. The PSCLC cell of Sample 1 was obtained, and its platelet texture was captured with a polarized optical microscope (POM, XPL-30TF, Shanghai WeiTu Optics and Electron Technology Co., Ltd., Shanghai, China) (Figure 3i). After the separation of the glass slides, the glass slide with PSCLC film was immersed in solvent of ethanol and water to wash out the unreactive components. When the proportion between ethanol and water was set as 7:1, the soaking time for the CLC template was 30 min or so. The stripped glass slide was again glued to the coated slide, which was heated on a hot stage to evaporate the solvent after the soaking process, under ultraviolet irradiation. The single-layer PSCLC template of Sample 1 was fabricated, and various optical characteristics could be integrated via refilling distinct materials into the template. Cell-1 was formed by refilling Sample-2 into the template from Sample-1, and Cell-2 was formd with refilled material of Sample-3 (Table 3). Cell-1 and Cell-2 were designed with fixed color in the visible band and adjustable reflection in the infrared band, respectively. POM images of Cell-1 and Cell-2 indicated that CLC domains of helical structure could be effectively reconstructed owing to the strong anchoring energy of the polymer template (Figure 3ii,iii). Considering the solar energy-dense region of over 1000 nm, Cell-1 and Cell-2 were fabricated to implement thermal management via the reflection band in and out of the solar energy-dense region. PSCLC of Sample-4 with reflection in the infrared band was fabricated (Figure 3iv), and the polymer template was obtained in terms of the fabrication process in Figure 2. Cell-3 was generated by refilling Sample-5 with reflection in the visible band into the PSCLC template from Sample-4 (Table 3). The surface morphology of Cell-3 indicated that the cholesteric phase could be effectively reconstructed after the refilling procedure (Figure 3v). Cell-3 was designed with various colors in the visible band and fixed reflection in the infrared band, showing dynamic color while shielding the infrared irradiation solar energy.

## 3. Thermal and Electrical Modulation on CLCs

**Figure 4:**
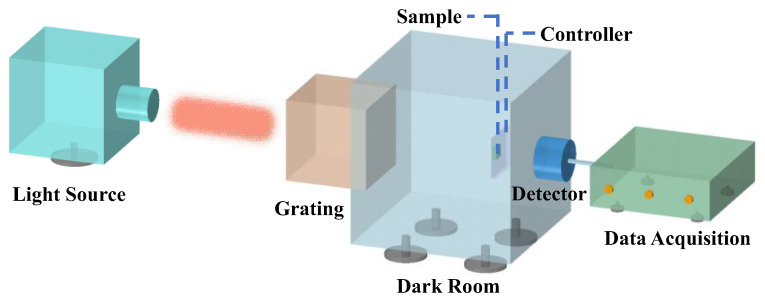
Experimental measurements of transmissions in the tunable single-layer templated CLC.

Optical performances of thermal and electrical modulation on two types of templated CLCs were characterized, respectively. Transmission spectra of the single-layer templated CLC covering the range from 400 nm to 1700 nm were measured using the system shown in Figure 4. In the experiment, unpolarized light from a tungsten bromine lamp was used as incident light. The temperature of samples was controlled using the hot stage, and an AC electric field was applied to samples with a function generator (AFG 3011, Tektronix, Beaverton, OR, USA). The optical response was detected using a data acquisition system (DCS300PA, Zolix, Beijing, China) connected to a computer. The transmittance was calculated as the ratio between detected light intensity with samples and that with an empty cell. Changes in transmission spectra induced by temperature and electric fields were also recorded on the system.

### 3.1. Optical Characteristics of Single-Layer Templated CLC with Adjustable Reflection in the Infrared Band

We fabricated single-layer templated CLCs—Cell-1 and Cell-2—of adjustable pitches in the infrared band and fixed reflections in the visible band for thermal control. The spectral characteristics of CLC cells upon application of an electric field and temperature were examined.

**Figure 5:**
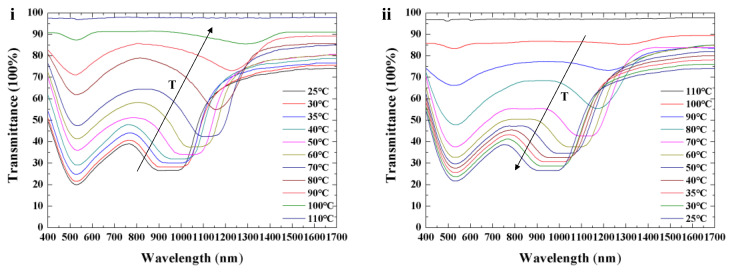
Optical properties of CLCs with adjustable reflection in the infrared upon heating (**i**) and cooling (**ii**) (T, temperature).

**Figure 6:**
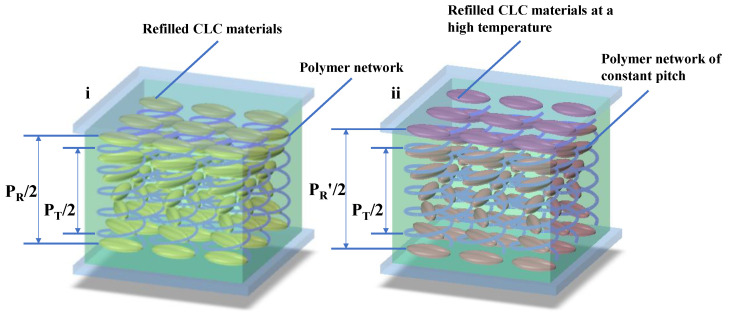
Pitch modulation of CLCs in a single-layer templated cell upon thermal activation. (**i**) Pitch of refilled material and template at initial temperature (P_R_, pitch of refilled material; P_T_, pitch of template). (**ii**) Longer pitch of refilled material and constant pitch of template at a high temperature (P_R_′, pitch of refilled material at a high temperature).

The transmission spectra shown in Figure 5 indicate simultaneous visible and infrared reflections from the cell. The spectral shifts of the two reflection bands as a function of temperature are visualized. Figure 5i illustrates transmission spectra of Cell 1 upon heating, and Figure 5ii shows transmission spectra of Cell 1 upon cooling. The reflection peak red shifts from 950 nm to 1305 nm when temperature increases from 25 °C to 100 °C. The transmittance decreases from 45% initially until the mixture reaches an isotropic state as the temperature increases. Temperature (T) dependence of pitch (P) [16,35,36] can be given by

(1) $$P=\frac{\lambda_{c}}{<n>\cdot cos\theta }$$

(2) $$\lambda_{c}=A(1+\frac{\beta }{T-T_{0}})$$

where $\lambda_{c}$ is the central wavelength of Bragg reflection, <n> is the average refractive index, θ is the angle between incident light and the normal (θ=0° in this work), A and β are molecular thermodynamic factors, and $T_{0}$ is the phase transition temperature. For the CLC material used in single-layer cell, the helical pitch increases with the increase in temperature ($\frac{dP}{dT}>0$). At 25 °C, the refilled material exhibits a flat and wide reflection band with a bottom width of 130 nm and full width at half-maximum of 320 nm in the infrared band, owing to the large birefringence of LCs and large cell gap. The center of the reflection band in the visible spectrum shifts little (1~2 nm as shown in Figure 5i) due to the strong interface anchoring energy of the polymer network from the template. The refilled material shows a flexible thermal response in the infrared, owing to little limitation from the polymer network, as shown in Figure 6. As temperature increases, the reflection band red shifts from 950 nm to 1305 nm (infrared region (>1000 nm), which irradiates a large amount of heat). The cell would be beneficial to the thermal management of buildings if applied in smart windows. Simultaneously, the cell showed a color in the visible region (at a central wavelength of 530 nm) induced by the template. As shown in Figure 5ii, the shift in the infrared band can be reversibly driven via heating and cooling.

**Figure 7:**
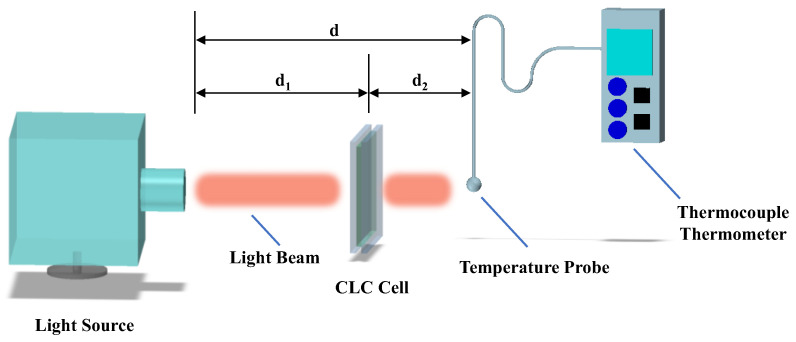
Temperature measurement diagram demonstrating thermal management effect of the single-layer templated CLC cell (d, distance between light source and temperature probe; d_1_, distance between light source and CLC cell; d_2_, distance between CLC cell and temperature probe).

**Figure 8:**
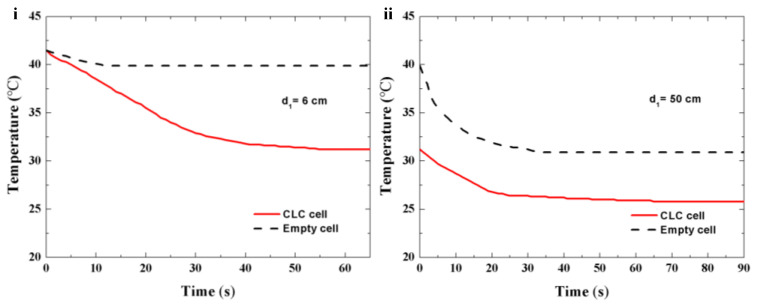
Temperature response curves of CLC cell and empty cell with d_1_ = 6 cm (**i**) and d_1_ = 50 cm (**ii**).

To evaluate the thermal management effect of the single-layer templated CLC cell Cell-1, a temperature measurement system was set up at room temperature (25 °C), as shown in Figure 7. The unpolarized broadband light from a tungsten bromine lamp (covering the range of 400 nm to 1700 nm) penetrated through the cells and then reached the temperature probe of the thermocouple thermometer (HT-9815, XINTEST, Shenzhen, China). The temperature variations in different states were recorded by the thermocouple thermometer. When the light beam directly reached the temperature probe without cells, with d = 8 cm, the steady temperature captured by the probe was 41.5 °C. When utilizing Cell-1 of fixed reflection in the visible band and adjustable reflection in the infrared band as a window, with d_1_ = 6 cm, d_2_ = 2 cm, the temperature decreased from 41.5 °C to 31.2 °C within 55 s (Figure 8i). After exposure with broadband light for 30 min, the temperature of the probe behind the CLC cell was stable at 31.2 °C. The temperature response of an empty cell, with d_1_ = 6 cm, d_2_ = 2 cm, was also measured. After the light beam penetrated an empty cell, the temperature decreased from 41.5 °C to 39.9 °C in 12 s (Figure 8i). Compared with an empty cell, the CLC cell showed an obvious cooling effect when the broadband light beam penetrated through the cell. To evaluate the effect of the CLC cell on potential energy savings for heating, the light source was moved away from the CLC cell along the optical axis direction, and then fixed at the position of d_1_ = 50 cm, d_2_ = 2 cm. The temperature variation in this process was recorded with the thermocouple thermometer, and the temperature response curves of the CLC cell and empty cell are illustrated in Figure 8ii. When the light source was moving away from the CLC cell, the temperature of the probe gradually decreased from 31.2 °C to 25.8 °C in 65 s. For the empty cell, the temperature of the thermocouple thermometer decreased from 39.9 °C to 30.7 °C within 39 s. Compared to the temperature effect of the empty cell, the CLC cell exhibited good heat preservation effect owing to the flexible shift of the reflection band in the infrared spectrum, keeping the temperature within a comfortable range for humans.

**Figure 9:**
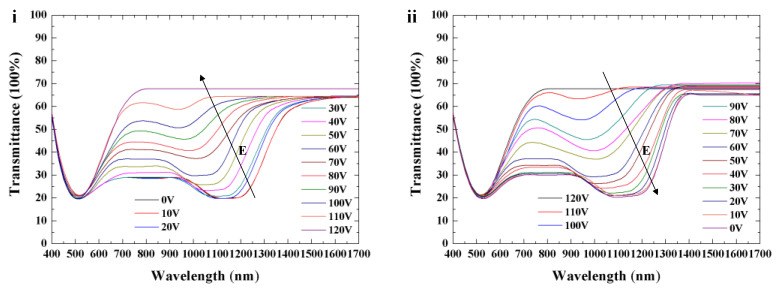
Optical characteristics of CLCs with adjustable reflection in the infrared band via increasing (**i**) and decreasing (**ii**) electric field (E, electric field).

**Figure 10:**
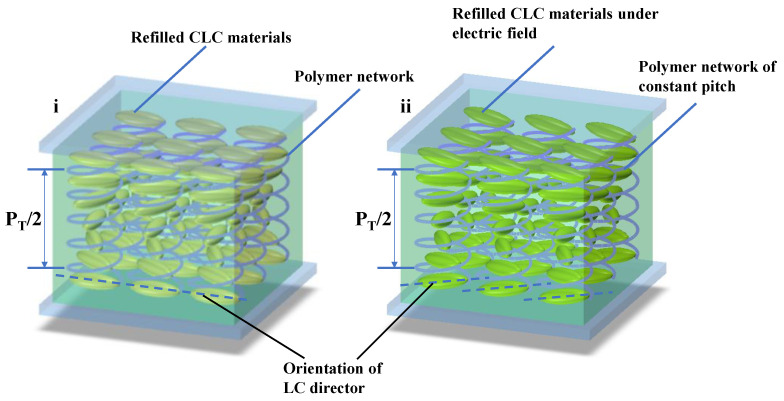
Molecular arrangements of CLC helix driven by electric field. (**i**) Lying molecules of refilled material and pitch of template without electric field (dashed lines indicate orientation of LC director). (**ii**) Tilted molecules of refilled material and constant pitch of template in an electric field.

In order to realize thermal management of a single-layer templated CLC via electric field, we fabricated Cell-2 by refilling Sample 3 into the template of Sample 1. The electro-optical properties of Cell-2 were investigated. A series of square-wave voltages with 1 kHz frequency were applied on the cell. As shown in Figure 9i, a blue shift in the position of the infrared reflection band from 1150 nm to 950 nm can be observed when the electric field increases from 0 V to 110 V (0 V/µm to 5.5 V/µm). Transmission spectra in the visible band are almost unchanged via electrical actuation. The transmittance of reflection band gradually decreases until the LC molecules align perpendicular to the substrate under the high electric field. A schematic diagram indicating the LC director orientation of Cell-2 via electric field is shown in Figure 10. The pitch length of the template remained approximately constant, owing to the ultra-high driving voltage of the polymer network from the template, while the LC directors of Sample 3 reoriented upon application of the electric field, resulting in the blue shift of the reflection band. For refilled CLC material, the critical field tilting LC molecules toward the field direction [37] is

(3) $$E_{c}=\frac{\pi }{h}\sqrt{\frac{K_{11}}{\varepsilon_{0}∆\varepsilon }}\cdot \sqrt{1+\frac{\left(K_{33}-2K_{22}\right)}{K_{11}}{\left(\frac{\Phi }{\pi }\right)}^{2}+2\frac{K_{22}}{K_{11}}(\frac{hq_{0}}{\pi })(\frac{\Phi }{\pi })}$$

(4) $$q_{0}=\frac{2\pi }{P}$$

where h is the cell gap, $q_{0}$ is chirality, $K_{11}$ is the splay elastic constant, $K_{22}$ is the twist elastic constant, $K_{33}$ is the bend elastic constant, Φ is the angle between two alignment directions, $\varepsilon_{0}$ is the permittivity of a vacuum, and ∆ε is the dielectric anisotropy. A threshold field $E_{P}$ introduced to describe the reorientation of the polymer template [37] can be given by

(5) $$E_{P}=\sqrt{\frac{\pi^{3}K}{(8+\pi^{2})\varepsilon_{0}∆\varepsilon }}\cdot \frac{\sqrt{c}}{R}$$

where c is the polymer concentration, R is the radius of the polymer fiber, and K is the assumption of the isotropic elastic constant ($K_{11}\approx K_{22}\approx K_{33}=K$). For the single-layer templated CLC cell, $E_{P}$ is far larger than $E_{c}$ due to the high density of polymer fibers, resulting in a relatively fixed reflection band in the visible spectrum. The shift in the infrared band can be reversibly actuated during the increase and decrease in the electric field, as shown in Figure 9.

The templated CLC with adjustable reflection in the infrared was capable of implementing automatic thermal control and active electric field regulation, which could contribute to energy conservation.

### 3.2. Spectral Properties of Single-Layer Templated CLC with Dynamic Color

**Figure 11:**
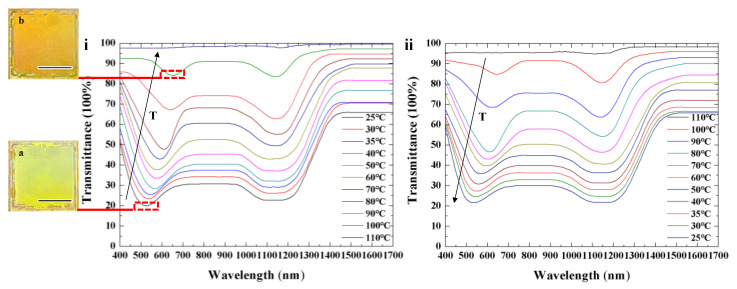
Transmission spectra of CLCs with dynamic color upon heating (**i**) and cooling (**ii**). The insets in (**i**) are photographs of Cell-3 showing dynamic color during modulation process. Scale bar, 1 cm.

The dependence of transmission spectra on temperature and electric field of single-layer templated CLCs with dynamic color was also investigated. The optical characteristics of Cell-3 during the heating and cooling process are illustrated in Figure 11. Simultaneous reflection bands in the visible (at an original central wavelength of 530 nm) and infrared (at a central wavelength of 1150 nm) spectra are achieved. The refilled material Sample 5 exhibited dynamic color with temperature variation. A color shift from 530 nm to 650 nm is achieved during thermal expansion, as shown in Figure 11i. The template of Sample 4 remains constant because of stabilization from the polymer network. The transmittance of the reflection band in the visible and infrared spectra gradually decreases until the mixture transforms into an isotropic phase. The central wavelength can be adjusted reversibly via thermal actuation, as illustrated in Figure 11. Cell-3 with a template in the infrared band and reflection in the visible band showed dynamic color during the heating process. In Cell-3, a greenish color was reflected at room temperature (inset image (a) in Figure 11i), while a red-colored reflection was obtained due to elongation of helical pitch at a high temperature (inset image (b) in Figure 11i).

**Figure 12:**
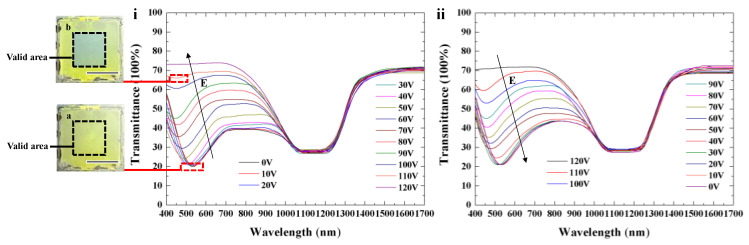
Spectral properties of CLCs with dynamic color upon increasing (**i**) and decreasing (**ii**) electric field. The insets in (**i**) illustrate the electric-field-induced color change of Cell-3 (the central square area was valid). Scale bar, 1 cm.

The spectral properties of Cell-3 as a function of the electric field were also investigated. Figure 12i shows a continuous blue shift from 530 nm to 440 nm with an increase in voltage from 0 V to 110 V. Transmission spectra in the infrared band of the template change little, owing to the strong anchoring force from the polymer network. The transmittance of the reflection band in the visible spectrum gradually decreases until the LC molecules orient perpendicular to the glass substrate. The central wavelength can be adjusted reversibly via electrical actuation, as illustrated in Figure 12. The electric-field-induced color change of Cell-3 in the original state (inset image (a) in Figure 12i) and in the electric field (inset image (b) in Figure 12i) was recorded. Initially, Cell-3 exhibited a greenish color without the influence of an external field. When an electric field was applied on Cell-3, the valid central square area showed a blue-colored reflection due to the rotation of LC directors induced by the electric field.

The spectrum plays an important part in biological clock regulation. For example, a blue crest between 460 nm and 480 nm was reported to show melatonin suppression effects, which was closely related to circadian rhythm regulation, and red light from 606 nm to 635 nm was likely to compensate for the melatonin suppression resulting from blue light [38,39,40]. The templated CLC with dynamic color was able to present automatic color change and active color adjustment, simultaneously blocking infrared radiation, which would be beneficial to biological clock regulation.

## 4. Conclusions

In summary, temperature self-adaptive and color-adjustable smart windows using templated CLCs are proposed. The smart window exhibited simultaneous reflections in the visible and infrared bands. Reversible reflection band shifts of CLCs can be induced upon thermal and electrical actuation. The position of the infrared reflection band can be adjusted from 950 nm to 1305 nm via temperature, and from 1150 nm to 950 nm via electric field in CLC with adjustable reflection in the infrared band. A temperature decrease of up to 10.3 °C within 55 s was induced by the single-layer templated CLC cell with adjustable reflection in the infrared band, and a comfortable temperature range was effectively maintained by the CLC cell in a varied environment. A color shift from 530 nm to 650 nm tuned by temperature and from 530 nm to 440 nm adjusted by electric field in the CLC with dynamic color in the visible spectrum were achieved. The templated CLC smart windows are suitable for applications particularly in energy conservation and biological clock regulation fields. However, the relatively low optical contrast in the visible spectrum and high driving electric field of the templated CLC smart window might be problematic due to the hazy and solid state of polymers in the templating process. In future work, the compositions and templating process would be optimized to achieve transparent and low driving voltage for the templated CLC smart window.

## Figures and Tables

**Table 1:** Samples for CLC cell of adjustable reflection in the infrared band.

Sample	HTD [wt%]	R811 [wt%]	TMPTA [wt%]	C3M [wt%]	IRG184 [wt%]
Sample-1	62.52	24.97	5.61	6.5	0.4
Sample-2	84.92	15.08			
Sample-3	87.77	12.23			

**Table 2:** Samples for CLC cell of dynamic color.

Sample	HTD [wt%]	R811 [wt%]	TMPTA [wt%]	C3M [wt%]	IRG184 [wt%]
Sample-4	78.21	10.91	6.20	4.29	0.39
Sample-5	71.45	28.55			

**Table 3:** Components of template and refilled material in CLC cells.

Cell	Template	Refilled Material
Cell-1	Sample-1	Sample-2
Cell-2	Sample-1	Sample-3
Cell-3	Sample-4	Sample-5

## Data Availability

Data are contained within the article.
